# Disentangling bilingualism and developmental language disorder in the acquisition of Spanish articles and clitics: Quantitative and qualitative contributions

**DOI:** 10.1017/S0305000925100354

**Published:** 2025-11-17

**Authors:** Patrick D. Thane, Anny Patricia Castilla-Earls, Alejandra Auza Benavides, Ana Teresa Pérez-Leroux

**Affiliations:** 1 Department of Spanish and Portuguese, The University of Texas at Austin, Austin, TX, USA; 2 Dual Lab, Department of Speech, Language, and Hearing, The University of Texas at Dallas, Dallas, TX, USA; 3 Hospital General Dr. Manuel Gea Gonzalez, Mexico City, Mexico; 4 Department of Spanish/Linguistics, University of Toronto, Toronto, ON, Canada

**Keywords:** developmental language disorder, bilingualism effects, Spanish as a heritage language, Trastorno de desarrollo del lenguaje, efectos de bilingüismo, español como lengua de herencia

## Abstract

This study explored the acquisition of Spanish nominal morphology in 116 children aged 4;0 to 6;11, grouped according to language ability (developmental language disorder [DLD] and typical development [TD]) and bilingualism (Spanish–English bilingual and Spanish monolingual). Monolinguals produced more target-like articles and direct object clitics than bilinguals, as did children with TD compared to peers with DLD. Bilinguals with TD produced more target-like morphology than monolinguals with DLD, particularly clitics. Children with DLD were more likely to omit clitics than peers with TD, but this contrast did not extend to bilinguals compared to monolinguals. Children produced singular default articles in plural contexts. Overall, our results suggest that clitics function better than articles for identifying DLD in bilinguals on quantitative and qualitative grounds.

## Resumen

Este estudio investigó la adquisición de la morfología nominal del español en 116 niños de edades 4;0 a 6;11, agrupados según su estatus clínico (trastorno del desarrollo del lenguaje, TDL y desarrollo típico, DT) y el bilingüismo (bilingüe en español e inglés, monolingüe en español). Los monolingües produjeron más artículos y clíticos de objeto directo anticipados que los bilingües, y los niños con DT produjeron más que los niños con TDL, especialmente con los clíticos. Los niños con TDL omitieron más clíticos que sus compañeros con DT, pero este contraste no se extendió a los niños bilingües en comparación con los monolingües. Los niños extendieron los artículos singulares a contextos del plural, lo que sugiere un uso por defecto del número en artículos. Los resultados sugieren que los clíticos funcionan mejor que los artículos en la identificación del TDL en niños hispanohablantes por razones tanto cuantitativas como cualitativas.

## 1. Introduction

Heritage languages (HLs) develop with limited exposure in a minority language context, where the input space is shared with a socially dominant language (Montrul, 2016; Polinsky & Kagan, 2007). Reduced input and constant interaction between languages often lead to a shift in dominance towards the majority language, which is a frequent phenomenon for Spanish speakers in the United States acquiring English upon entering school (Castilla-Earls et al., 2019; Hiebert & Rojas, 2021). These dominance shifts often lead to bilingualism effects (see Pirvulescu et al., 2014), causing reduced input in the HL and crosslinguistic influence from the majority language. Bilingualism effects can lead to structural differences in (Montrul, 2008, 2013; Putnam & Sánchez, 2013) and/or protracted development of (e.g. Martinez-Nieto & Restrepo, 2022; Solano-Escobar & Cuza, 2023; Thane, 2024, 2025) the HL grammar. The area of grammar most impacted by these effects is inflectional morphology, due to its interdependence with syntax, semantics, and the lexicon. In this way, inflectional morphology represents the bottleneck of HL acquisition (Montrul, 2018). This area of grammar is also a bottleneck for children with developmental language disorder (DLD), both bilingual and monolingual (Bishop, 2017), and the morphosyntactic patterns of children with DLD often resemble those of bilinguals.

The similarities in language production between Spanish heritage speakers (HSs) with typical development (TD) and Spanish-speaking children with DLD lead to frequent overdiagnosis of DLD in bilinguals, at rates of up to 40% (Bonuck et al., 2022). Overdiagnosis can divert important but limited clinical resources away from children in true need of intervention. Moreover, and equally importantly, misdiagnosis transmits a negative sociolinguistic message that bilingual varieties require remediation despite emerging under typical circumstances of language contact. Therefore, establishing which morphosyntactic structures can distinguish between TD and atypical development in bilinguals, as well as monolinguals, is a question of linguistic justice. Answering such a question stands to establish appropriate norms of typical bilingual development where widespread support for multilingualism may not be available.

To date, studies on the acquisition of morphosyntax by Spanish-speaking children have generally compared either bilinguals to monolinguals or children with DLD to those with TD. Early insights into this topic stem from Morgan et al’s (2009) study, which demonstrated that Spanish articles, clitics, subjunctive mood, and derivational morphology could not differentiate bilinguals with TD from monolingual children with DLD, although an overall comparison combining all structures did yield group-level differences (see also Gutiérrez-Clellen et al., 2008 for a similar approach in English). By using a similar research design, we aim to identify methods for identifying DLD in both bilingual and monolingual contexts. In so doing, our study is well-positioned to recognize what is truly an instance of DLD within the linguistic variation that is characteristic of heritage Spanish, which aligns with Oetting’s (2018) and Oetting et al.’s (2016) *disability within difference* framework. However, we argue that including monolingual data alongside bilingual participants to sort out bilingualism effects from DLD has two advantages. Firstly, this design allows us to identify possible patterns that can be used to diagnose atypical development regardless of bilingualism. Relatedly, it enables us to identify which phenomena in linguistic development are unique to contexts of bilingualism, which are components of (a)typical development in all children, and which are common to both.

The present study builds on extensive research evaluating the acquisition of Spanish articles and direct object clitics by coupling quantitative analyses of production rates with a qualitative analysis of patterns of non-target responses. While *quality* and *qualitative* are generally used in bilingualism research to refer to the input that children receive, we usethese terminologies here to refer to the nature of what children produce. On clinical grounds, understanding what children with typical versus atypical development produce in place of target-like inflectional morphology (i.e. the qualitative nature of their responses) could prove to be as important in identifying DLD as how much target-like inflectional morphology they produce. However, generally, children’s response patterns are reviewed descriptively. Consequently, we explore both differences in rates of target-like production (quantity) and nature of responses (quality) of articles and clitics in Spanish as a potential method for distinguishing between children with DLD and TD.

## 2. Structure and acquisition of Spanish nominal morphology

Both articles and clitics, the structures considered here, are components of the agreement system of Spanish, which has inflections in the nominal domain for gender and number. With inanimate nouns, gender is lexically assigned (animate referents, however, are not assigned arbitrarily but are not addressed here; see Kramer, 2015). However, number is determined by the quantity of the noun. Therefore, Spanish grammatical gender is lexically assigned to nouns, while number is computed through context rather than lexically.

Within the noun phrase, Spanish articles are marked for definiteness, gender, and number, and adjectives carry inflections for the latter two categories to agree with their nominal referent. Spanish also features direct object clitic pronouns, which are phonologically and syntactically dependent on verbal hosts. Clitics are produced preverbally in simple verb phrases and can be realized preverbally or postverbally in complex verb phrases, depending on the first verb. Third-person direct object clitics are also marked for both the gender (masculine or feminine) and number (singular or plural) of their nominal referent, resulting in four possible forms. Clitic gender and number are often vital for disambiguating between referents in the discourse.

English does not have grammatical gender, nor does it have number agreement with determiners and adjectives.^1^ Number, however, is realized on third-person direct object pronouns in English (e.g. *it* versus *them*). Therefore, agreement requires the computation of both gender and number features in Spanish, but only a number feature on pronouns in English, pointing to possible crosslinguistic influence. Table 1 lists the Spanish articles and third-person direct object clitics and their gender and number.

**Table 1.** Summary of Spanish articles and direct object clitics by gender and number

Number	Masculine	Feminine
Singular	*el* (definite article) *un* (indefinite article) *lo* (direct object clitic)	*la* (definite article) *una* (indefinite article) *la* (direct object clitic)
Plural	*los* (definite article) *unos* (indefinite article) *los* (direct object clitic)	*las* (definite article) *unas* (indefinite article) *las* (direct object clitic)

Research carried out within multiple theoretical frameworks has noted that masculine gender is the unmarked form often used as a default in place of feminine agreement morphology (e.g. Beatty-Martínez & Dussias, 2019; Harris, 1991; McCarthy, 2005, 2008). McCarthy’s (2005, 2008) Morphological Underspecification Hypothesis provides a systematic account for defaults and links the overextension of certain morphological forms such as masculine or singular to underspecification in the syntax. Following her account, situated within the Distributed Morphology approach (e.g. Embick & Noyer, 2007; Halle & Marantz, 1993), masculine and singular categories do not require gender or number projections in the syntax. Consequently, they may surface as defaults (or *elsewhere forms*) in feminine or plural contexts because they are less specified, but the opposite effect, in which overspecified forms such as feminine or plural surface in place of masculine or singular ones, is not expected. This proposal was originally advanced for adult bilinguals, yet might be useful for child HL development and could even be applicable to children with DLD, who experience persistent difficulties with agreement (e.g. use of forms such as *el casa*, ‘the house’, instead of *la casa*; see Rice & Wexler, 1996). For instance, if children with DLD substitute overspecified forms (e.g. feminine for masculine or plural for singular), but bilingual children who have TD do not, overuse of feminine gender in masculine contexts or plural forms in singular contexts could be an indicator of atypical bilingual development.

Before continuing, it is useful to consider the possible non-target responses that bilingual children may provide. In addition to codeswitched, English-only, or unintelligible responses, the terms *substitution* and *omission* are frequently used in the literature to account for response patterns, as described at length below. At the word level, omissions occur when no article or clitic is produced in an expected context, and substitutions occur when a non-target form is used in place of a target one (e.g. for articles, *el*, masculine singular, instead of *la*, feminine singular). However, within Distributed Morphology, which holds that syntactic processes are involved below the level of the lexical item, not producing plural agreement, such as substituting *lo* for *los*, could be considered an omission of the number projection. To be consistent with previous research, in our study, we refer to the use of a masculine article or clitic with feminine nouns as a gender default and the use of a singular article or clitic with plural nouns as a number default, both of which we counted as substitutions. However, we recognize that a singular default could constitute omission within the Distributed Morphology framework because plural marking is not affixed to the singular base form.

### 2.1. Articles

Monolingual Spanish-speaking children with TD acquire gender agreement in the noun phrase between ages three and four (e.g. Hernández Pina, 1984; Mariscal, 2009). Several studies have demonstrated that bilingual and monolingual Spanish-speaking children with DLD make considerably more substitutions (i.e. use of a non-target form in place of a target one) and omissions of articles than TD peers (Bedore & Leonard, 2001, 2005; Castilla-Earls et al., 2016, 2020a, 2021b; Jacobson, 2012; Morgan et al., 2009; Restrepo & Gutiérrez-Clellen, 2001). This resonates with observations reported in a previous study (Auza Benavides, 2009), which could provide further context for understanding substitutions and omissions, especially when viewed through a semantic lens. These observations have generally been descriptive in nature and document that children with DLD often make near-miss article substitutions, whereby they produce articles that agree in *either* gender *or* number, as well as omissions, when not using target-like forms (e.g. Bedore & Leonard, 2001, 2005; Jacobson, 2012). Both masculine substitutions for feminine and feminine substitutions for masculine have been attested in monolinguals with DLD (Bedore & Leonard, 2005). Such a finding implies bidirectional substitution of gender (i.e. masculine to feminine and feminine to masculine) that is not documented in typical HL development, yet statistical evidence would be helpful to further substantiate these claims. This, in turn, would have clinical and theoretical implications: On a clinical level, bidirectional gender substitution could be a useful indicator of DLD independent of bilingualism effects. On a theoretical level, this result would suggest that the Morphological Underspecification Hypothesis (McCarthy, 2005, 2008) does not apply to DLD populations.

The acquisition of gender agreement in the noun phrase by child HSs of Spanish has received considerable attention. HSs with lower exposure to Spanish or earlier ages of acquisition of English extend masculine morphology to feminine contexts (Anderson, 2001; Cuza & Pérez-Tattam, 2016; Montrul & Potowski, 2007). The reverse effect is far less frequent, which supports McCarthy’s (2005, 2008) Morphological Underspecification Hypothesis. Article omission has also been documented in research on adult (Montrul & Ionin, 2012) and child (Cuza et al., 2025) Spanish HSs in generic plural noun phrases due to semantic crosslinguistic influence from English. Therefore, since bilinguals both substitute and omit articles, they may appear qualitatively similar to children with DLD, complicating diagnosis. Number agreement has not yet been directly researched with Spanish HS children, perhaps underscoring an assumption that this morphological category would be less susceptible to crosslinguistic influence given its clearer semantic contribution and its instantiation on English nouns.

Therefore, there are three open questions on the acquisition of articles in Spanish. Firstly, it is unclear whether the directionality of substitutions that has been demonstrated for HSs’ acquisition of gender extends to children with DLD. Secondly, while monolingual children with DLD as well as bilinguals have exhibited similar substitution and omission tendencies across previous research, it is unclear precisely how similar these tendencies are without a comparison of monolingual and bilingual children with DLD and TD in the same study. Finally, little is known about the acquisition of number agreement. Exploring these questions stands to improve our ability to characterize the systematic innovations of US Spanish (i.e. difference), identify patterns representative of DLD in Spanish HSs (i.e. disorder within difference), and reveal markers of atypical development that serve both bilingual and monolingual populations.

### 2.2. Direct object clitics

Previous findings suggest that monolingual Spanish-speaking children with TD reach adult-like clitic production as early as age four (Eisenchlas, 2003), although Castilla and Pérez-Leroux (2010) reported optional clitic use by 5-year-old children. On quantitative grounds, lower target-like production rates of clitics are a well-established identifier of DLD in Spanish-speaking children (e.g. 11; Bedore & Leonard, 2001, 2005; Bosch & Serra, 1997; Castilla-Earls et al., 2020a, 2021b; Gutiérrez-Clellen et al., 2006; Jacobson, 2012; Morgan et al., 2009), as well as in French (e.g. Paradis et al., 2006) and Italian (e.g. Guasti et al., 2016). At the qualitative level, clitic omission is the most frequent type of non-target response for DLD children. In cases of substitution, children with DLD often exhibit “near misses,” similar to articles. Morgan et al.’s (2009, p. 589) findings demonstrate more accurate production of masculine singular than feminine singular clitics, as well as greater accuracy with singular than plural clitics, in monolingual and bilingual children with DLD. Bedore and Leonard (2001) also reported singular clitic defaults for plural nouns, but documented bidirectional gender substitution in monolingual children with DLD. These findings are predominantly descriptive, so statistical evidence could be useful for building on these claims.

Research on child Spanish HSs points to an overextension of masculine clitics to feminine contexts (Austin et al., 2024; Shin et al., 2019), particularly by children with higher receptive vocabulary in English (Shin et al., 2019), supporting McCarthy’s (2005, 2008) default account; however, alternative evidence also exists that did not report a masculine default in spontaneous production (Montanari et al., 2024). Shin et al. (2019, 2022) also demonstrated that HSs with lower Spanish lexical proficiency and fewer experiences using the HL were more likely to omit objects or to use pragmatically infelicitous (yet grammatical) lexical noun phrases instead of clitics. Therefore, some HSs with TD may appear similar to children with DLD, further complicating diagnosis. Lastly, like with articles, clitic number agreement has not been analysed in contexts of HL development.

To summarize, both language ability and bilingualism effects influence rates and patterns of target clitic production. Clitic omission is a well-attested phenomenon in Spanish-speaking children with DLD. Some bilinguals with TD appear to substitute or even omit clitics as well, which makes identifying DLD in contexts of bilingualism particularly challenging. Studies on bilinguals with TD also report overextension of masculine gender to feminine contexts. Understanding whether children with DLD substitute unidirectionally (predominantly masculine in place of feminine) or bidirectionally (both genders substituted for one another) may hold promise in disentangling TD and atypical development in bilinguals (i.e. disability within difference; Oetting, 2018, Oetting et al., 2016). Moreover, little is known about number in the acquisition of nominal agreement, with both clitics and articles.

## 3. The study

Because there is not yet a study that distinguishes between bilinguals and monolinguals with DLD and TD at the group level that also considers the nature of differences between these groups’ non-target production patterns statistically, three research questions were proposed:

1. Are there quantitative differences between bilingual and monolingual children with and without DLD in their production rates of Spanish articles and direct object clitics?

We anticipated that monolinguals would produce more target-like articles than bilinguals of the same language ability (i.e. bilinguals with TD > bilinguals with DLD and monolinguals with TD > monolinguals with DLD) and that children with DLD would do so more than peers with TD. In line with Morgan et al. (2009), we predicted that bilingual children with TD would produce more target-like morphology than monolingual peers with DLD in a combined statistical model evaluating both articles and clitics, but not in models for each structure assessed individually.

1. Are there qualitative differences between bilingual and monolingual children with DLD and TD in the distribution of forms that they produce in place of target articles and clitics?

We anticipated that children with DLD would produce more omissions of articles and clitics than their TD peers, in line with previous research (e.g. Bedore & Leonard, 2001, 2005; Jacobson, 2012). We also anticipated more substitutions, rather than omissions, in the use of articles and clitics among bilinguals compared to monolinguals. Finally, we anticipated that bilingual children with DLD would produce more omissions than substitutions. Such a finding could pinpoint omissions as a clinical marker of DLD in bilinguals, compatible with a disability within difference context (Oetting, 2018; Oetting et al., 2016).

1. Are there morphological defaults for masculine gender and singular number in the production of nominal morphology by children with DLD and/or who are bilingual?

Based on previous research (e.g. Austin et al., 2024; Cuza & Pérez-Tattam, 2016; Montrul & Potowski, 2007), we anticipated more target-like use of masculine than feminine gender by bilinguals. Although there is limited previous evidence, we predicted the same pattern for children with DLD, given the previous descriptions in Morgan et al. (2009) and Bedore and Leonard (2001). However, given that number is also realized on English nouns and pronouns, we did not predict a default for singular number for bilingual children, nor for those with DLD. Such findings would support McCarthy’s (2005, 2008) Morphological Underspecification Hypothesis for gender, given its crosslinguistic differences from English and its lower interpretability with inanimate referents, but not for number, both in bilingual children and in those with DLD.

## 4. Methods

### 4.1. Participants

A total of 116 Spanish-speaking children participated in this study (68 boys and 48 girls). Sixty-six Spanish–English bilingual children from the United States, whose data are reported in Castilla-Earls et al. (2021b), and 50 monolingual children from Mexico, whose data are reported in Castilla-Earls et al. (2020a), were analysed here. The bilingual children were from northwestern New York (*n* = 36) and Houston, Texas (*n* = 30). Forty-eight children (72%) received free and reduced-price lunch. Mothers of 20 children had obtained a primary education, 18 had completed high school, 4 had some college education, 20 held a professional or university degree, and education level was not available for 4. In addition, 79% of mothers spoke mostly or only Spanish with their children, 17% reported using both languages, and 2% spoke mainly English at home. Children were from multiple Spanish-speaking regions: 30 families reported Mexico as their family’s origin, 15 were from Puerto Rico, 1 was from Guatemala, 2 reported being from the mainland United States, and 13 reported “other” as their origin. Data on origin was not available for five families. Among the children, 33 met the inclusion criteria for DLD described below and comprised the bilingual DLD (BL-DLD) group (average age 57.5 months), and 33 children formed the bilingual TD (BL-TD) group (average age 65.1 months).

All bilingual children were in preschool, kindergarten, or first-grade classrooms at the time of data collection. Children from upstate New York attended English-only schools, and Spanish was not the dominant language of their local community. In contrast, all children in Houston, Texas, had access to transitional bilingual education programmes. Their schools were located in predominantly Latinx neighbourhoods where there was a higher prevalence of Spanish in the surrounding community. Despite these differences in the sociolinguistic communities, in Castilla-Earls et al. (2021b), origin (New York versus Texas) did not emerge as a significant factor in statistical modelling, and as such, we do not include it as a covariate here.

The monolingual children were from Mexico City. Among them, 25 had been classified as DLD and comprised the monolingual DLD (ML-DLD) group (average age 64.9 months), and 25 comprised the monolingual TD (ML-TD) group (average age 68.2 months). Mothers of 19 children had obtained a primary education, 17 had completed high school, 6 had some college education, 5 held a professional or university degree, and educational data was not available for 3. Participants’ average age across all groups was 63.6 months; due to differences in average age between groups, centred age in months was included as a predictor in statistical modelling.

To be considered for participation, all children passed a hearing screening and obtained a standard score above 70 on the nonverbal intelligence subsection of the *Kaufman Brief Intelligence Test* (Kaufman & Kaufman, 2004). Classification as DLD relied on two metrics for each child: the cutoff standardized score from the morphosyntax subtest on the Bilingual English-Spanish Assessment (BESA; Peña et al., 2014) and the percentage of grammatical utterances (PGU) on a spontaneous oral retell. The BESA is widely used in the diagnosis of DLD in bilingual populations and offers sensitivity above 90% and specificity above 80%. Bilingual children completed the BESA in Spanish and English, and their best score was considered. The BESA indicates DLD cutoff scores in the stronger language of 84 for children aged 4;0–4;11, 85 for children aged 5;0–5;11, and 81 for children aged 6;0–6;11. While the BESA is designed for diagnosing DLD in bilingual children, for purposes of consistency, monolingual children completed the Spanish morphosyntax subsection, and their score was also used to classify these participants as having DLD or TD. For the second metric, obtaining below 80% PGU on the spontaneous language sample in both languages was considered indicative of DLD for bilinguals; monolingual children with a Spanish PGU below 80% were similarly classified as having DLD.

### 4.2. Tasks

As stated above, all children completed the nonverbal intelligence subtest on the KBIT (Kaufman & Kaufman, 2004), as well as the BESA morphosyntax subtest. Additionally, children completed a story retell task based on *Frog, Where Are You?* (Mayer, 1980), from which PGU was calculated with which to identify children with DLD. Children completed this retell after the research assistant told a scripted version of the same story. The retells were recorded and subsequently transcribed, and PGU was computed using the Systematic Analysis of Language Transcripts (Miller & Iglesias, 2014) software. Children completed additional standardized measures omitted from this manuscript since their data are not considered here.

Finally, to address research questions, all participants completed the *Desarrollo morfosintáctico del español* (DEME; Castilla-Earls et al., 2021a), a picture-based elicited production task. Eight items elicited articles: three masculine singular, one feminine singular, two masculine plural, and two feminine plural. Six of these items were definite articles and two were indefinite. Ten additional items elicited direct object clitics: three for masculine singular *lo*, three for feminine singular *la*, two for masculine plural *los*, and two for feminine plural *las.* Fifteen additional items tested verbal morphology; their results are discussed in separate projects. This task followed a contextualized sentence completion format, where children heard a preamble based on two pictures and needed to answer questions about the second picture that were conducive to the use of target structures.

We acknowledge that the DEME task contains an uneven number of items across gender and number combinations, as well as across articles and clitics. We aimed to be consistent with previous studies (Castilla-Earls et al., 2021a), who, in the validation of the DEME task, removed items that showed low reliability among all groups of children due to below-chance target-like production rates. This is particularly evident with feminine singular articles, in which only one item showed sufficient reliability to be included in our tasks. Our motivations not to include these items were twofold. Firstly, we aimed to be consistent with previous studies that have used the DEME task (e.g., Castilla-Earls et al., 2016, 2019, 2020a, 2020b, 2021a, 2021b). Secondly, we believed that including these items, which showed unexpectedly low target-like production rates, could produce misleading results comparing children with DLD and TD on the one hand and bilinguals and monolinguals on the other.

### 4.3. Procedure

Data collection took place between 2014 and 2017, with approval from the Institutional Review Boards of Hospital General Dr. Manuel Gea González (Mexico City), the State University of New York Fredonia, and the University of Houston. Following parental consent and child assent, children completed between two and three 50-minute sessions in their school or at a location designated by caretakers. Bilingual data collection took place across three sessions, and languages were kept separate across these sessions. Monolinguals completed two sessions.

## 5. Results

**Figure 1.**
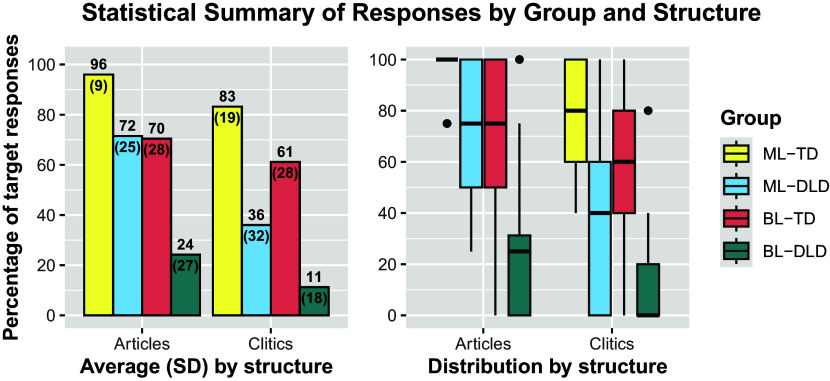
Summary of production by group and structure, reported as percentages.^2^

Data were analysed in RStudio (R Core Team, 2022) using the *broom.mixed* (Bolker & Robinson, 2018), *lme4* (Bates et al., 2015), *lmerTest* (Kuznetsova et al., 2017), *patchwork* (Pedersen, 2024), and *tidyverse* (Wickham et al., 2019) packages. Anonymized data and code were made available on a public GitHub repository (https://github.com/pthane/DEME-Bilingualism-Analysis). The binary dependent variable was production of target-like morphology on the DEME task: participants received a response score of *1* for providing the target article or clitic and *0* for any other response. Unintelligible utterances, use of English, and responses in which target morphology was omitted were scored as 0. The independent variables were group (BL-DLD, BL-TD, ML-DLD, and ML-TD), bilingualism (bilingual and monolingual), and language ability (DLD and TD). ^3^ Centred age in months was a continuous predictor. The target-like production rates of articles and clitics by group are summarized in Figure 1.

### 5.1. Multivariate analyses

Generalized linear mixed effects binary logistic regression models were conducted to examine response score as a function of group and centred age in months as predictors, with participant and item as random effects. Three sets of models were prepared: the combined set incorporated data across both structures, the articles set evaluated articles only, and the clitics set evaluated clitics only. Each set contained four models, one with each of the groups (ML-TD, ML-DLD, BL-TD, and BL-DLD) as reference level. The results of these models are provided in Appendix 1. For ease of interpretation, we converted all log odds to probabilities of target-like production. These probabilities are represented here as percentages. Each percentage can be interpreted as the likelihood of a particular group producing the target-like structure. Lower percentages indicate a lower likelihood of target-like production, while percentages closer to 100% imply a higher likelihood of the production of a target-like structure. The formula for the conversion of log odds to probabilities, where *p* represents the probability of a target-like response as a percentage, is prob = (exp(log odds)/[1 + (exp(log odds))] * 100).

To summarize the data in Appendix 1, children with TD produced more target morphology than peers with DLD (i.e. BL-TD > BL-DLD and ML-TD > ML-DLD). Similarly, monolingual children produced more target morphology than bilingual peers with the same language ability across sets (i.e. ML-DLD > BL-DLD and ML-TD > BL-TD). Clitics and the combined model revealed statistically significant differences between BL-TD and ML-DLD groups at the *p* < .05 level, but this contrast was not evident for articles. As children grew older, they produced more target-like morphology across all sets.

### 5.2. Differences in substitution and omission rates

**Figure 2.**
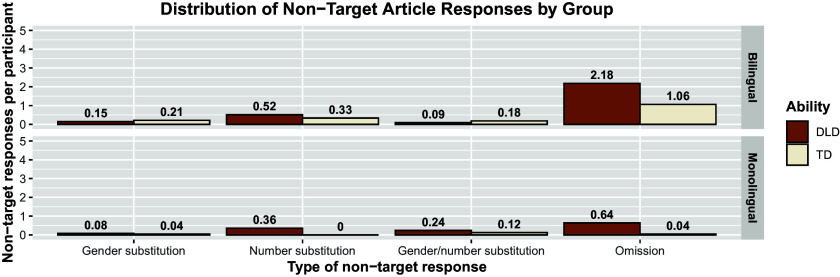
Distribution of non-target responses for articles by group per participant.

**Figure 3.**
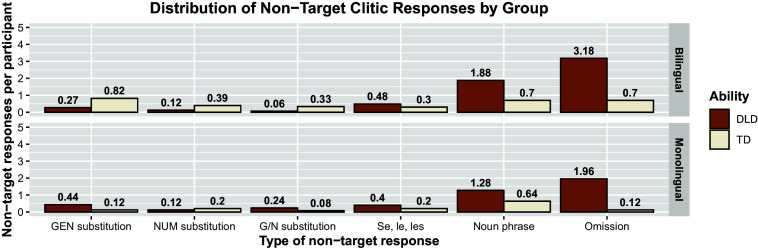
Distribution of non-target responses for clitics by group per participant.^4^

***Descriptive findings.*** To complement these findings, we carried out an analysis of children’s alternative responses for articles and clitics, targeting qualitative differences that explore what specific groups of children produce as alternatives to target-like morphology. Responses in English and those that were unintelligible were not analysed, as they did not allow us to recognize patterns of non-target morphology in Spanish. For articles, non-target responses were divided into four categories: gender substitutions, number substitutions, gender and number substitutions, and omissions. Figure 2 represents how frequently each type of non-target response for articles occurred in each group, divided by the number of participants in that group. This accounted for differences in group size and provided an average for each substitution type within each group. For clitics, non-target responses were divided into four categories: direct object clitic gender substitutions, direct object clitic number substitutions, direct object clitic gender and number substitutions, substitutions of other types of clitics (*le*, *les*, and *se*),^5^ noun substitutions, and omissions. The patterns of non-target clitic responses by group are summarized in Figure 3. As in Figure 2, the values in Figure 3 represent the average number of each type of non-target response for clitics per participant within their group. Like with articles, this approach accounted for differences in group size.

Figure 2 indicates that for articles, omission was the most common non-target response pattern for the ML-DLD group and both bilingual groups. Single substitutions (gender *or* number) were more common than double substitutions (gender *and* number) for all groups, as in previous research. Substitution and omission of articles for the ML-TD group were fleetingly rare. Figure 3 indicates that for clitics, gender substitutions were the most common type of non-target production for BL-TD children, while omissions and the use of lexical noun phrases were far more common for both groups of children with DLD. As with articles, double substitutions were documented but were less than half as frequent as single substitutions. It is also noteworthy that the most common clitic substitution for the BL-DLD group involved the use of the clitics *se*, *le*, and *les*, a point that we address further in the discussion.

***Differences in distributions of substitutions and omissions.*** To determine whether omissions and substitutions are more common in situations of bilingualism and/or DLD, two additional sets of two generalized linear mixed effects binomial logistic regressions (four in total) were carried out. All substitution categories from Figures 2 and 3 were assigned a score of 1 and merged into a single variable, substitution, such that a score of 0 represented omission. The models included only non-target production data with substitution as the independent variable and participant and item as random effects. There were two models for articles, one incorporating language ability (TD as reference level) as the predictor and the other with bilingualism (monolinguals as reference level) as the predictor. An additional two models prepared for clitics followed the same structure. The results of each model are shown in Appendix 2. As with the group-level models, we converted log odds to probabilities of substitutions, reported here as percentages.^6^

For articles, there was no statistically significant difference in substitutions between children with DLD and TD (*p* = .206); however, the difference between bilinguals and monolinguals (*p* = .034) was significant. Bilingual children had a probability of .240 of producing a substitution, suggesting that 24% of non-target responses involved using a non-target article, while monolinguals had a probability of .565 of substituting a non-target form. Therefore, despite overall fewer non-target responses than bilinguals, when considering non-target productions only, monolinguals had a significantly higher proportion of substitutions than bilinguals. Relatedly, nearly all substitutions of articles by monolingual children occurred with the ML-DLD group, but substitutions occurred with both groups of bilinguals.

For clitics, there were statistically significant differences in substitution (*p* < .001) between children with DLD and those with TD. Children with DLD had a probability of substitution of .047, suggesting that they substitute clitics in only 4.7% of non-target responses, while children with TD had a probability of .861, suggesting that 86.1% of non-target responses were substitutions. Consequently, children with DLD omitted far more clitics, a difference that was significant, and children with TD rarely did so. However, the contrast between bilingual and monolingual children was not significant (*p* = 0.563). Therefore, clitic omissions were significantly more likely in children with DLD than in those with TD but not more likely in bilinguals than in monolinguals.

### 5.3. Substitution patterns

**Figure 4.**
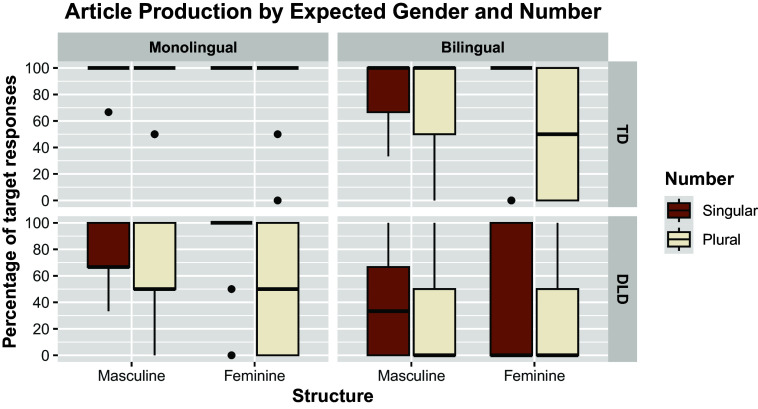
Article production by group, gender, and number. Averages are summarized through squares.

**Figure 5.**
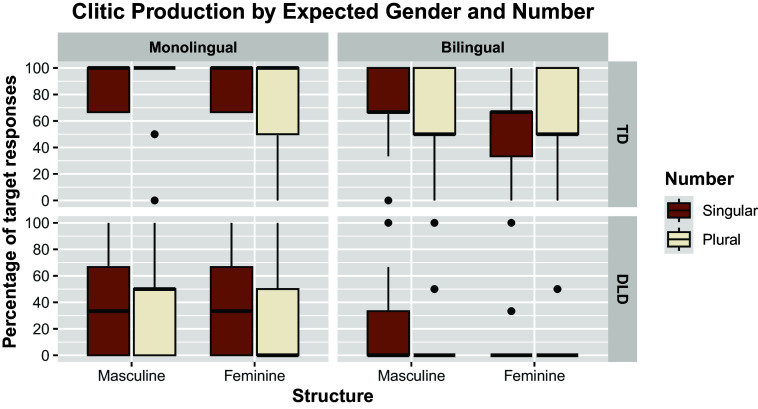
Clitic production by group, gender, and number.

To better understand the nature of substitution patterns and whether they constitute evidence of gender and number defaults, a final set of four generalized linear mixed effects binomial logistic regressions was necessary. The first two models addressed the role of gender and number, respectively, with articles, and the latter two addressed the same with clitics. Each model integrated response score as the binary dependent variable as well as participant and item as random effects. The expected gender of the article or clitic (masculine item as reference level, feminine), bilingualism (monolinguals as reference level, bilinguals), and language ability (TD as reference level, DLD) were included as fixed effects for gender models; number models followed a similar structure, replacing gender with the expected number (singular item as reference level, plural). Interactions between expected gender/number and bilingualism as well as between expected gender/number and language ability were also incorporated. The distribution of responses by group, expected gender, and expected number is shown in Figure 4 for articles and Figure 5 for clitics. The results of these models are given in Appendix 3.

There was no significant effect at the *p* > .05 level that favoured the extension of masculine gender to expectedly feminine contexts with articles, but children were more likely to produce target-like articles in expectedly singular contexts than plural ones. In contrast, there were no significant effects at the *p* > .05 level for masculine gender in expectedly feminine contexts or singular number in expectedly plural contexts with clitics. All models reaffirmed the effects of bilingualism and DLD on the production of target morphology. However, we observed a tendency favouring the target-like use of masculine over feminine clitics in children with DLD in Figure 5. Therefore, we conducted a final binomial logistic regression post-hoc exploring response score as a function of expected gender, with participant and item as random effects, using data from participants with DLD only. There was no significant effect for masculine gender (*β* = 0.48, SE = 0.32, *p* = .139).

### 5.4. Individual differences

**Figure 6.**
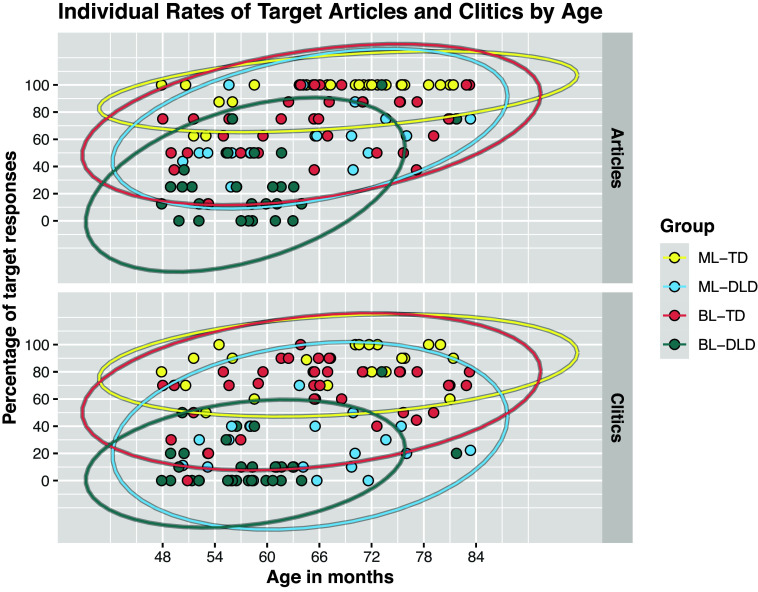
Individual rates of target-like article and clitic production by age in months and group.

Finally, we present individual rates of target-like article and clitic production in Figure 6 as a function of age in months and group. Since there were different numbers of stimuli for articles and clitics, we report target-like production as percentages for ease of comparison. We point out six insights that these data provide that could not be captured in group-level analyses. Firstly, there were participants in all four groups who produced categorically target-like articles; for clitics, eight children, seven in the ML-TD group and one in the BL-TD group, did so. Secondly, some children in the BL-DLD, BL-TD, and ML-DLD groups produced target-like articles and clitics within the range of ML-TD participants. These observations support the notion that the group-level trends do not apply to all individual HSs who have myriad linguistic experiences impacting their exposure to and use of Spanish, nor to all children who experience atypical development. Thirdly, and relatedly, for both articles and clitics, there are spaces where the four circles representing group-level trends overlap, suggesting that the upper bounds of the BL-DLD group, comprised of participants who experience both bilingualism effects and DLD, are within the range of the lower bounds of the ML-TD group. For articles, this overlap occurs between approximately 51 and 75 months and 75% and 85% target-like production. For clitics, this overlap occurs between approximately 51 and 72 months and 50% and 60% target-like production. Therefore, individual patterns may not align with overall group-level trends, and even the two groups that are contrasted in two ways – in both their bilingualism and language ability – can overlap, despite sizeable group-level differences in the statistical modelling and descriptive data.

Fourthly, we note that there is far higher variation in target-like production rates in the youngest than in the oldest children: there were children 48 months old (4;0) in the BL-DLD group that produced 0% target-like inflection, but there were no participants of the maximum 83 months old (6;11) who did so. Fifthly, most children alternated between target and non-target production of articles and especially clitics. Therefore, most children show evidence of having acquired the syntax of article and clitic placement and agreement, even if they alternate between target-like and non-target forms. Finally, there are children with DLD who produced articles and especially clitics in a more target-like fashion than peers with TD, such that group-level trends in language ability do not categorically extend to all children. Each of these insights raises questions as to how to account for such individual variability that are beyond the scope of this paper, as we discuss subsequently, but we aim to inspire future research exploring these variables in greater detail.

### 5.5. Summary

To summarize, our results revealed significant differences between bilingual and monolingual groups of the same language ability, as well as between DLD and TD groups with the same language profile (i.e. bilingual or monolingual), in the combined, articles, and clitics sets. The BL-TD group produced significantly more target morphology than the ML-DLD group in the nominal and clitics sets, but not articles. Analyses of substitution and omission patterns for articles did not reveal a significant difference between children with DLD and TD, although monolinguals produced more substitutions than bilinguals. Children with DLD were more likely to omit clitics than those with TD, but we did not find the same contrast between bilingual and monolingual children. Therefore, clitic omissions could be a predictor of DLD both within bilingual communities and independently of the number of languages spoken. There was evidence of default singular forms for plural articles across groups. An analysis of individual differences reaffirms the effects of group and age in the acquisition of articles and clitics, demonstrating some bilingual children pattern with monolinguals and some individuals with DLD pattern within the range of peers with TD. Most children alternated between producing target and non-target forms.

## 6. Discussion

The present study explored the effects of language ability and bilingualism in the acquisition of Spanish nominal morphology by children aged 4;0 to 6;11. We aimed to tease apart TD from atypical development in child bilinguals with Spanish as a HL. Although we explored both bilingual and monolingual data, our study can contribute to establishing markers of DLD within the context of US Spanish, a goal that is consistent with the disorder within difference approach (e.g. Oetting, 2018; Oetting et al., 2016). However, we also incorporated monolingual data as a point of comparison and to provide insights into the diagnosis of DLD regardless of bilingualism. We evaluated the rates and response patterns of Spanish articles and clitics with four groups of children: bilinguals with DLD, bilinguals with TD, monolinguals with DLD, and monolinguals with TD. We explored both quantitative and qualitative differences between groups to expand upon previous findings. We first address our three research questions, and subsequently, we discuss the implications of our findings for bilingualism theory and clinical practice, before closing with limitations for future work.

Our first research question addressed differences in target-like production rates of articles and clitics between groups. We predicted differences between DLD and TD groups and between bilingual and monolingual groups, but we anticipated that only the combined set of regressions would be able to detect differences between ML-DLD and BL-TD groups. As anticipated, TD groups produced the expected morphology more frequently than DLD groups with the same language profile (i.e. BL-TD > BL-DLD and ML-TD > ML-DLD) in the combined, articles, and clitics sets. These findings are consistent with numerous previous studies demonstrating that nominal morphosyntax represents an area of considerable acquisitional difficulty for Spanish-speaking children with DLD in both bilingual and monolingual contexts (e.g. Bedore & Leonard, 2001, 2005; Castilla-Earls et al., 2020a, 2021b; Jacobson, 2012; Morgan et al., 2009). Monolingual children produced more expected morphology than bilinguals of the same language ability (i.e. ML-DLD > BL-DLD and ML-TD > BL-TD) in all sets. Our findings differ subtly from Morgan et al. (2009), who did not detect structure-specific differences between BL-TD and ML-DLD groups. The finding that clitics differentiated between typical bilingual and atypical monolingual development in this study is likely due to our larger sample size, which allowed us to detect these differences more clearly. However, both studies were consistent in that articles alone cannot distinguish between typical contexts of bilingualism and atypical monolingual development. A logical clinical implication of this research is that clitics, rather than articles, may prove more useful in distinguishing between what is typical and what represents a clinical need regardless of bilingualism.

Our second research question examined whether bilingual and monolingual children with and without DLD differed in the types of non-target responses they produced. We hypothesized that children with DLD would make more article and clitic omissions than their peers with TD, while bilinguals would produce more gender substitutions than monolinguals. Children with DLD produced significantly more omissions than their TD peers for clitics, but not for articles. Monolinguals substituted articles at a significantly higher rate than bilinguals, yet the ML-TD group had very high rates of target-like production. Therefore, when monolinguals did not produce target-like responses, their proportion of substitutions was higher than for bilingual children; however, bilingual children produced more non-target morphology overall. These findings partially support our hypothesis regarding the higher rate of omissions among children with DLD, but only for clitics. However, contrary to our hypothesis, bilinguals did not substitute articles and clitics more than monolinguals. Once again, clitic omissions can distinguish between DLD and TD independently of bilingualism, unlike articles.

Our third and final research question addressed whether there was evidence of morphological defaults for gender and number in the production of article and clitic morphology for children with DLD and/or who were bilingual. We hypothesized that there would be masculine gender and singular number defaults observable for these groups, given that they are underspecified forms that require fewer agreement projections. Our data showed no significant effect for masculine gender in either structure for bilinguals, but did reveal an effect for singular number morphology with articles. This absence of a masculine default in Spanish HSs is consistent with Montanari et al.’s (2024) study with preschool-aged children, but not with Cuza and Pérez-Tattam’s (2016) and Montrul and Potowski’s (2007) studies, which assessed school-aged children. A possibility is that gender defaults emerge later in development, as the latter two studies incorporated older participants. More data with a broader age range is needed to explore this possibility.

We turn now to broader discussions of the implications of our data for bilingualism theory and clinical practice. Firstly, as captured in the findings for our third research question, our data provide some support to McCarthy’s (2005, 2008) Morphological Underspecification Hypothesis. Figure 3 suggests that children were more likely to substitute gender *or* number than to substitute both, suggesting that these features are calculated through separate projections in the syntax and that defaults are separate for both. For articles, plural number appears to be more difficult for children to acquire regardless of DLD or bilingualism, suggesting that all children may pass through a period of underspecification for number that extends into the age range tested. In addition to substituting singular and masculine clitics, children, particularly bilinguals with DLD, substituted other types of clitics, including *se*, which does not vary for gender or number, as well as the dative (indirect object) clitics *le* (her/him) and *les* (them), which are marked for number only. These clitics involve no or only one agreement feature and are thus less specified than direct object clitics that have both gender and number features. Therefore, the use of singular number with articles and of less-specified clitics suggests the use of underspecified forms, consistent with McCarthy’s (2005, 2008) account originally proposed for bilinguals. This pattern is consistent with Austin et al. (2024), who reported frequent use of *se* for direct object clitics among child HSs, and invites the possibility that morphological underspecification is not limited to gender and number and may also be amenable to contexts other than the acquisition of the less-dominant language, such as the acquisition of morphosyntax by children with DLD.

Secondly, we turn to the implications of our work for bilingualism and HL acquisition. We argue that quantitative differences between bilinguals and monolinguals, while part of our hypotheses, need not be interpreted as an “advantage” or “disadvantage” for monolinguals. Rather, they should be considered differences in development when bilingual children have a variety of exposure patterns to Spanish at home, school, and the community in comparison with monolinguals who are only exposed to one language, all the time and in all contexts. Our observations of bilinguals only concern one of the two languages that these children speak and, as such, are only intended to represent part of their linguistic development. Consequently, it is desirable to describe this difference in developmental patterns so that bilingual children are not incorrectly penalized when only comparing them to monolingual benchmarks.

The positive correlation between target-like production and age in months in all our statistical modelling demonstrates that bilingual children between ages 4;0 and 6;11 continue to acquire Spanish nominal morphology, albeit at a slower rate, than monolinguals. This is no small feat considering that these children are mastering multiple languages with diverging nominal agreement systems simultaneously. These findings are consistent with existing research on Spanish (e.g. Martinez-Nieto & Restrepo, 2022; Solano-Escobar & Cuza, 2023) and other HLs (Daskalaki et al., 2023; Jia & Paradis, 2020; Torregrossa et al., 2023; Thane, 2024, 2025), suggesting that some bilinguals follow a path of protracted morphosyntactic development: at an age range when monolinguals have mastered articles but continue to master clitics, Spanish HSs continue to master both structures. Critically, the individual analyses highlight that bilinguals do not categorically differ from monolinguals and that many bilingual children were within the range of monolinguals of the same language ability in their target-like production of articles and clitics. Most bilingual children did not categorically omit nor categorically produce target-like articles or clitics, but rather alternated between target and non-target forms in their production. Consequently, we do not conclude that most Spanish HSs have not acquired the syntax of articles or clitics, but that additional factors not captured here may account for the within-speaker variation observed.

We also note that in the article data, the emergence of a default for number across groups, but not for gender, suggests that all children may pass through a period of morphological underspecification, and it is highly plausible that child HSs move through this same stage in their development later on or for a longer period of time due to bilingualism effects. Therefore, we aim to highlight that bilingual children continue to master agreement into the early school years, and, regarding number with articles, are exhibiting the same pattern as monolingual children. Consequently, it would be inaccurate to claim that bilinguals follow a different developmental path compared to monolinguals, even if they move along this path at a slower rate than peers who are tasked with acquiring only one language.

On a clinical level, we argue that clitics are of greater value in identifying DLD in bilingual populations on quantitative and qualitative grounds. Firstly, both TD groups produced significantly more clitics than the groups of children with DLD. Secondly, children with DLD were more likely to omit clitics than TD children, but this same contrast did not extend to the comparison between bilingual and monolingual children. Neither of these patterns extended to articles, even though articles appear to be faster for children to acquire (Castilla-Earls et al., 2020b; Shin et al., 2019) and are less vulnerable to attrition (Goebel-Mahrle & Shin, 2020) than clitics in child HSs. However, the use of a singular article in a plural context may not be a sign of atypical development in children aged between 4;0 and 6;11, because we observe this pattern independent of DLD and bilingualism effects. Our data strongly underscore the need for using metrics that are normed on bilingual populations in making clinical decisions, as Bedore and Peña (2008) advocate and as emphasized in disability within difference approaches (Oetting, 2018; Oetting et al., 2016). For instance, since bilingual children with TD appear quantitatively similar to monolinguals with DLD in their rates of target-like articles, assessments using this structure that do not take bilingualism into consideration could lead to the false conclusion that typically developing bilinguals need intervention. As we have argued, flagging typical bilinguals as in need of speech-language services has pejorative implications that can be damaging to bilingual identities and that drain resources from bilingual children in true need of speech-language services.

Before concluding, we hope to address some limitations of our study that may inspire future work. Firstly, we cannot determine whether the quantitative or qualitative differences exhibited here are attributable to processing or representational causes in the absence of receptive data. Future studies may wish to integrate receptive tasks to complement findings in production. An additional limitation is that elicited production limits freedom in language selection, and previous findings have reported more target-like use of Spanish inflectional morphology in spontaneous than in elicited production (Bedore & Leonard, 2001, 2005). As Pirvulescu (2006) states, there are advantages and drawbacks of both types of production data. Therefore, an ideal future direction would be to triangulate spontaneous and elicited production data with receptive data. We do, however, highlight that our particular task was somewhat open in nature since children were asked to respond to a prompt freely, rather than to complete a partially completed sentence.

On one hand, it is simpler to identify non-target forms or establish a denominator of target-like production with elicitation tasks. Spontaneous tasks possess openness that enable variability in structures children used, which may make it harder for researchers to pinpoint specific areas that may be useful in the diagnosis of DLD or in documenting typical bilingual development. On the other, however, the openness of spontaneous production offers a valuable insight into how children process and express meanings using alternative forms to the target response. This flexibility reveals not only their grammatical knowledge but also their strategies for handling processing loads and coping with linguistic demands. Since children with DLD also experience lower working memory and are more affected by greater processing loads (Larson & Ellis Weismer, 2022), structural distance could have accounted for between-participant variation, although this is beyond the purview of our experiment. Similarly, bilinguals dominant in English may experience a greater processing load when producing articles and clitics, particularly if utilized in lengthy sentences. Future studies could possibly account for individual differences in working memory and for structural distance in analyses, both welcome contributions that would build beyond the scope of the present study.

Moreover, to maintain consistency with other studies using the same task (e.g. Castilla-Earls et al., 2016, 2020a, 2020b, 2021a, 2021b), we excluded items eliciting articles and clitics that showed low reliability. Therefore, children had fewer opportunities to use feminine than masculine gender, and there were more stimuli eliciting clitics than articles. Consequently, future studies may wish to triangulate elicited and spontaneous data using a consistent number of items across genders and numbers.

Additionally, to be consistent with previous work that has provided qualitative descriptions of children’s non-target production of article and clitic morphology (e.g. Bedore & Leonard, 2001, 2005; Morgan et al., 2009), we categorized any use of one lexical item (such as a feminine singular clitic) in place of another (such as a feminine plural clitic) as a substitution. This approach is logical on clinical grounds, as it does not require clinicians to become familiar with morphological theory in which the undersuppliance of plural morphology can be interpreted as an omission. However, theories of Distributed Morphology (e.g. Embick & Noyer, 2007), such as the very one assumed by McCarthy (2005, 2008), do not hold privileged status for the lexical item, such that syntactic operations apply to sub-lexical processes such as plural formation. Therefore, the lack of plural marking on a singular clitic with a plural referent could be reanalysed as equivalent to an omission under this theory in future work.

Finally, our findings are primarily comprised of group-level analyses, but bilingualism is a largely individual phenomenon. We recognize that individual experiences with the HL shape their acquisition, as emphasized by Putnam and Sánchez’s (2013) model concerning the acquisition of HL morphosyntax, and that individual patterns of exposure are a critical factor to explore in greater detail in future work. Children with lower exposure may experience greater bilingualism effects, which could make it particularly challenging to disentangle: a bilingual child with TD and low HL exposure could plausibly perform similarly to a Spanish-dominant bilingual with DLD. DLD and TD. While work investigating individual differences in the context of DLD has remained elusive in general (see Paradis, 2023 for a discussion of existing studies), we acknowledge that individual differences is a critical component of accounting for HSs in our findings.

Despite these limitations, our study makes novel contributions in multiple ways. The primary contribution of our findings is that clitics allow us to distinguish between typical and atypical bilingual development, including between monolinguals with DLD and children who are typically developing bilinguals. Our results also demonstrated that clitic omissions were an indicator of DLD for HSs as well as for monolinguals. Neither of these findings applied to articles, such that clitics may hold greater promise in identifying DLD in both bilingual and monolingual populations. However, we do note greater target-like production of singular than plural articles. Moreover, we contribute to a growing body of work showing protracted development in child HSs’ morphosyntactic systems. We aim for our work to highlight the complex developmental trajectory of Spanish nominal morphology in young bilingual children and to set the stage for future research, both within the HL acquisition and/or DLD literature, considering how best to account for the patterns of individual-level variation that we observed.

## Footnotes

1 Number interacts with determiner use, since English allows bare subjects with plural count nouns and singular mass nouns. For example, *elephants are big* generically refers to all elephants through a bare plural noun subject. English also allows bare singular mass noun subjects, as in the sentence *sugar is not good for you.*

2 Percentages are more appropriate for descriptive statistics than the number of correct responses because each grammatical structure had a different number of items on the production task.

3 Note that models that incorporated group as the four-level distinction between BL-DLD, BL-TD, ML-DLD, and ML-TD never incorporated language ability and/or bilingualism, as this would lead to multicollinearity.

4 GEN implies gender substitution, NUM implies number substitution, and G/N implies gender and number substitution.

5
*Le* and *les* are indirect object clitics and *se* is a reflexive, passive, and aspectual clitic.

6 Note that since only those responses with substitutions and omissions were reported, any response that was not a substitution was an omission and vice versa. Thus, the probability of participants making an omission is the probability of making a substitution subtracted from 1. Estimates for omissions are the inverse of those for substitutions, with the same significance and standard error.

## Appendix 1. Generalized linear mixed effects modelling for group and age

**Table:**

Effect	Estimate	Probability (%)^7^	SE	*p*	Reference	Set
*Intercept*	2.47	92.2	0.00	*< .001*	ML-TD	Overall
ML-DLD	−2.56	47.7	0.00	*< .001*	ML-TD	Overall
BL-TD	−1.58	71.0	0.00	*< .001*	ML-TD	Overall
BL-DLD	−4.10	16.4	0.00	*< .001*	ML-TD	Overall
Age in months	0.05	95.3	0.00	*< .001*	ML-TD	Overall
*Intercept*	−0.09	47.7	0.00	*< .001*	ML-DLD	Overall
ML-TD	2.56	92.2	0.00	*< .001*	ML-DLD	Overall
BL-TD	0.98	70.9	0.00	*< .001*	ML-DLD	Overall
BL-DLD	−1.53	16.4	0.00	*< .001*	ML-DLD	Overall
Age in months	0.05	61.1	0.00	*< .001*	ML-DLD	Overall
*Intercept*	0.89	71.0	0.00	*< .001*	BL-TD	Overall
ML-TD	1.58	92.2	0.00	*< .001*	BL-TD	Overall
ML-DLD	−0.99	47.7	0.00	*< .001*	BL-TD	Overall
BL-DLD	−2.53	16.4	0.00	*< .001*	BL-TD	Overall
Age in months	0.05	80.8	0.00	*< .001*	BL-TD	Overall
*Intercept*	−1.63	16.4	0.00	*< .001*	BL-DLD	Overall
ML-TD	4.10	92.2	0.00	*< .001*	BL-DLD	Overall
ML-DLD	1.54	47.7	0.00	*< .001*	BL-DLD	Overall
BL-TD	2.53	71.0	0.00	*< .001*	BL-DLD	Overall
Age in months	0.05	25.2	0.00	*< .001*	BL-DLD	Overall
*Intercept*	3.64	97.4	0.55	*< .001*	ML-TD	Articles
ML-DLD	−2.68	72.3	0.54	*< .001*	ML-TD	Articles
BL-TD	−2.51	75.6	0.53	*< .001*	ML-TD	Articles
BL-DLD	−4.60	27.7	0.57	*< .001*	ML-TD	Articles
Age in months	0.07	98.9	0.02	*< .001*	ML-TD	Articles
*Intercept*	0.96	72.3	0.39	*.014*	ML-DLD	Articles
ML-TD	2.68	97.4	0.54	*< .001*	ML-DLD	Articles
BL-TD	0.18	75.6	0.38	.642	ML-DLD	Articles
BL-DLD	−1.92	27.7	0.40	*< .001*	ML-DLD	Articles
Age in months	0.07	85.7	0.02	*< .001*	ML-DLD	Articles
*Intercept*	1.13	75.6	0.37	*0.002*	BL-TD	Articles
ML-TD	2.51	97.4	0.53	*< .001*	BL-TD	Articles
ML-DLD	−0.18	72.3	0.38	.642	BL-TD	Articles
BL-DLD	−2.09	27.7	0.39	*< .001*	BL-TD	Articles
Age in months	0.07	87.7	0.02	*< .001*	BL-TD	Articles
*Intercept*	−0.96	27.7	0.38	*.013*	BL-DLD	Articles
ML-TD	4.60	97.4	0.57	*< .001*	BL-DLD	Articles
ML-DLD	1.92	72.3	0.40	*< .001*	BL-DLD	Articles
BL-TD	2.09	75.6	0.39	*< .001*	BL-DLD	Articles
Age in months	0.07	46.9	0.02	*< .001*	BL-DLD	Articles
*Intercept*	1.94	87.5	0.33	*< .001*	ML-TD	Clitics
ML-DLD	−2.91	27.5	0.40	*< .001*	ML-TD	Clitics
BL-TD	−1.23	67.1	0.37	*< .001*	ML-TD	Clitics
BL-DLD	−4.20	9.5	0.44	*< .001*	ML-TD	Clitics
Age in months	0.03	91.3	0.01	*.014*	ML-TD	Clitics
*Intercept*	−0.97	27.5	0.30	*.001*	ML-DLD	Clitics
ML-TD	2.91	87.5	0.40	*< .001*	ML-DLD	Clitics
BL-TD	1.68	67.1	0.35	*< .001*	ML-DLD	Clitics
BL-DLD	−1.29	9.5	0.40	*.001*	ML-DLD	Clitics
Age in months	0.03	36.4	0.01	*.014*	ML-DLD	Clitics
*Intercept*	0.71	67.1	0.26	*.006*	BL-TD	Clitics
ML-TD	1.23	87.5	0.37	*.001*	BL-TD	Clitics
ML-DLD	−1.68	27.5	0.35	*< .001*	BL-TD	Clitics
BL-DLD	−2.97	9.5	0.38	*< .001*	BL-TD	Clitics
Age in months	0.03	75.4	0.01	*.014*	BL-TD	Clitics
*Intercept*	−2.25	9.5	0.33	*< .001*	BL-DLD	Clitics
ML-TD	4.20	87.5	0.44	*< .001*	BL-DLD	Clitics
ML-DLD	1.29	27.5	0.40	*.001*	BL-DLD	Clitics
BL-TD	2.97	67.1	0.38	*< .001*	BL-DLD	Clitics
Age in months	0.03	13.7	0.01	*.014*	BL-DLD	Clitics

## Appendix 2. Generalized linear mixed effects modelling for substitutions

**Table:**

Effect	Estimate	Probability (%)^8^	SE	*p*	Reference	Contrast
*(Intercept)*	−0.21	44.8	0.40	.597	Articles	TD/DLD
DLD status	−0.64	34.5	0.51	.206	Articles	TD/DLD
*(Intercept)*	0.26	56.5	0.47	.582	Articles	BL/ML
Bilingual lang. group	−1.15	24.0	0.54	*.034*	Articles	BL/ML
*(Intercept)*	1.82	86.1	0.48	< .001	Clitics	TD/DLD
DLD status	−3.02	4.7	0.63	< .001	Clitics	TD/DLD
*(Intercept)*	0.27	56.7	0.50	0.582	Clitics	BL/ML
Bilingual lang. group	−0.35	41.3	0.61	0.563	Clitics	BL/ML

## Appendix 3. Generalized linear mixed effects modelling for substitution pattern

**Table:**

Effect	β	SE	p	Model
(Intercept)	3.472	0.614	*< .001*	
Masculine gender	0.593	0.687	.388	
Bilingual	−2.694	0.437	*< .001*	Article gender
DLD	−2.59	0.426	*< .001*	
Masculine gender : bilingual	0.218	0.42	.604	
Masculine gender : DLD	−0.247	0.413	.549	
(Intercept)	3.036	0.451	*< .001*	Article number
Singular number	1.725	0.555	*.002*	
Bilingual	−2.33	0.391	*< .001*	
DLD	−2.676	0.388	*< .001*	
Singular number : bilingual	−0.574	0.428	.180	
Singular number : DLD	−0.116	0.411	.778	
(Intercept)	2.02	0.338	*< .001*	
Masculine gender	0.29	0.383	.454	
Bilingual	−1.61	0.327	*< .001*	Clitic gender
DLD	−3.15	0.339	*< .001*	
Masculine gender : bilingual	0.35	0.335	.295	
Masculine gender : DLD	0.01	0.336	.983	
(Intercept)	1.86	0.37	*< .001*	Clitic number
Singular number	0.53	0.41	.200	
Bilingual	−1.25	0.338	*< .001*	
DLD	−3.02	0.35	*< .001*	
Singular number : bilingual	−0.32	0.338	.339	
Singular number : DLD	−0.22	0.339	.527	
